# Severe Blunt Muscle Trauma in Rats: Only Marginal Hypoxia in the Injured Area

**DOI:** 10.1371/journal.pone.0111151

**Published:** 2014-10-31

**Authors:** Kristina Funk, Nina Scheerer, Rabea Verhaegh, Carolin Pütter, Joachim Fandrey, Herbert de Groot

**Affiliations:** 1 University of Duisburg-Essen, Institute of Physiological Chemistry, University Hospital Essen, Essen, Germany; 2 University of Duisburg-Essen, Institute of Physiology, University Hospital Essen, Essen, Germany; 3 University of Duisburg-Essen, Institute for Medical Informatics, Biometry and Epidemiology, University Hospital Essen, Essen, Germany; Duke University Medical Center, United States of America

## Abstract

**Background:**

After severe muscle trauma, hypoxia due to microvascular perfusion failure is generally believed to further increase local injury and to impair healing. However, detailed analysis of hypoxia at the cellular level is missing. Therefore, in the present work, spectroscopic measurements of microvascular blood flow and O_2_ supply were combined with immunological detection of hypoxic cells to estimate O_2_ conditions within the injured muscle area.

**Materials and Methods:**

Severe blunt muscle trauma was induced in the right *Musculus gastrocnemius* of male Wistar rats by a standardized “weight-drop” device. Microvascular blood flow, relative hemoglobin amount, and hemoglobin O_2_ saturation were determined by laser Doppler and white-light spectroscopy. Hypoxic cells were detected by histologic evaluation of covalent binding of pimonidazole and expression of HIF-1α.

**Results:**

Directly after trauma and until the end of experiment (480 minutes), microvascular blood flow and relative hemoglobin amount were clearly increased. In contrast to blood flow and relative hemoglobin amount, there was no immediate but a delayed increase of microvascular hemoglobin O_2_ saturation. Pimonidazole immunostaining revealed a hypoxic fraction (percentage area of pimonidazole-labelled muscle cells within the injured area) between 8 to 3%. There was almost no HIF-1α expression detectable in the muscle cells under each condition studied.

**Conclusions:**

In the early phase (up to 8 hours) after severe blunt muscle trauma, the overall microvascular perfusion of the injured area and thus its O_2_ supply is clearly increased. This increased O_2_ supply is obviously sufficient to ensure normoxic (or even hyperoxic) conditions in the vast majority of the cells.

## Introduction

Traumatic muscle injury is generally believed to go along with immediate hypoxia within the injured area [1]–[6]. It is assumed that tissue injury further increases and healing is disturbed due to the deficiency of O_2_. Hypoxia is considered as a logical consequence of an impaired microvascular perfusion resulting from mechanically destroyed blood vessels, edema formation with increased tissue pressure, and vasoconstriction due to a sympatho-adrenergic response, being further aggravated by external and internal blood loss and shock. In line with these considerations, a decrease in functional capillary density of traumatically injured muscles has been demonstrated by several intravital fluorescence microscopy studies [6]–[11]. Increases in NAD(P)H fluorescence [9], [11], [12] and decreases in reduction of triphenyltetrazolium chloride (TTC) [13], both reflecting a restriction of oxidative mitochondrial metabolism, likewise suggest hypoxia within the injured muscle area.

On the other hand, there is clear evidence for an increased macrovascular blood supply to the injured area [14]–[16], and even an increased O_2_ content of the draining venous blood has been reported [17], [18]. Stimulated by these apparent discrepancies, we started the present project to study microvascular perfusion and O_2_ supply at the microvascular and microscopic level within a traumatically injured muscle area. We chose a rat model with a standardized “weight-drop” device causing a severe blunt muscle trauma of the *Musculus gastrocnemius* without any major blood loss, fracture or overt systemic reactions. To analyze O_2_ supply and O_2_ conditions, we combined measurements of microvascular perfusion and hemoglobin O_2_ saturation by laser Doppler and white-light spectroscopy, respectively, with the microscopic evaluation of hypoxic areas by the immunological determination of the covalent binding of pimonidazole and of the expression of the hypoxia-inducible α-subunit of the transcription factor HIF-1.

## Materials and Methods

### Ethics Statement

Experiments were conducted in accordance with the standards of Annex III of the directive 2010/63/EU of the European Parliament and of the Council of 22 September 2010 on the protection of animals used for scientific purposes [19]. The experimental protocol was reviewed and approved by the local Animal Care and Use Committee (Animal Care Center, University of Duisburg-Essen, Essen, Germany, and the district government of Düsseldorf (“North Rhine-Westphalia State Environment Agency”, Recklinghausen), Germany) with a Permit Number 8.87–50.10.37.09.254, G1076/09. All surgery was performed under isoflurane anaesthesia, and all efforts were made to minimize suffering.

### Animals

Male Wistar rats (421–470 g) were obtained from the central animal unit of the Essen University Hospital. Animals were kept under standardized conditions of temperature (22±1°C), humidity (55%±5%), and 12-h/12-h light/dark cycles with free access to food (Ssniff-Spezialdiäten, Soest, Germany) and water. All animals received humane care according to standards of Federation of European Laboratory Animal Science Association (FELASA).

### Chemicals

Paraffin (Paraplast Tissue Embedding Medium REF 501006) was purchased from McCormick Scientific (St. Louis, MO) and hematoxylin from Merck (Germany). Hypoxyprobe-^TM^1-Kit was obtained from HPI (Burlington, USA). Protein block and polyclonal rabbit anti-mouse immunoglobulins were from DAKO (Denmark). H_2_O_2_ was from Roth (Germany), Tween 20 from Sigma-Aldrich (Germany), ABC kit from Vector Laboratories (Burlington, USA), 3′,3′-diaminobenzidine (DAB) from Thermo Fisher Scientific (Fremont, USA), and mouse anti-HIF-1α antibody from Novus Biologicals (Cambridge, UK). Isoflurane (Florene) was obtained from Abbott (Wiesbaden, Germany), ketamine 10% from Ceva (Düsseldorf, Germany), lidocaine (Xylocain 1%) from AstraZeneca (Wedel, Germany), and Ringer's solution Macoflex N from MacoPharma International (Langen, Germany). Portex catheters (0.58 mm i.d., 0.96 mm o.d.) were purchased from Smiths Medical International (Hythe, UK) and medical oxygen was from Air Liquide (Düsseldorf, Germany).

### Procedure of blunt muscle trauma

Anaesthesia, analgesia as well as surgical and trauma procedure were basically performed as described previously [20] with minor modifications:

Rats were anaesthetized with isoflurane through face masks connected to a vaporizer (Dräger Medical, Lübeck, Germany). Depending on the group studied, induction of anaesthesia was performed with 2% isoflurane in 100% or 21% medical O_2_ at 4.0 l/min. During the experiment isoflurane was reduced to 1,5%–2% with a fresh gas flow of 1.0 l/min. All animals received ketamine (50 mg/kg body weight, s.c.) into the right chest wall for analgesia. After local lidocaine application (5 mg/kg body weight, s.c.) a median skin-deep incision was made along the throat and Portex catheters were placed within the left carotid artery and within the right jugular vein, respectively. The carotid artery cannula was used for continuous monitoring of systolic and diastolic blood pressure, mean arterial pressure and heart rate, while volume substitution (with sterile 0.9% NaCl solution; 5 ml/kg body weight per hour) was performed via the jugular vein cannula. The intraluminal location of the catheters was verified by blood aspiration before they were fixed by surgical suture. Subsequently, both lower hind limb were shaved. The overall preparation time before trauma induction was 60 minutes, including 20 minutes without any intervention, allowing the animals to adapt. Throughout the experiment, the depth of narcosis and analgesia was frequently controlled by pain reflexes, arterial blood pressure, blood gas analyses, and pulse oximetry, and regulated accordingly. Body temperature was maintained at 37°C using a temperature-controlled heating pad and covering the animals with aluminium foil.

Before the induction of trauma, the right lower hind limb was positioned in a retainer to avoid breakage of the bones and hind limb movement during the impact. Trauma was applied to the right *Musculus gastrocnemius* using a standardized “weight-drop” device with a 1-kg weight (impact area, 2×2 cm) falling from 37 cm, thus resulting in an energy density of 0.9 J/cm^2^. This energy density ensured that after trauma skin was intact and no blood loss was visible. In addition no bone fracture was apparent. Produced severe muscle injury was histologically indicated by disrupted muscle cells, formation of edema and hemorrhage.

At the end of the experimental time, both gastrocnemius muscles and overlying skin were excised and the rats were sacrificed by cardiac incision under deep isoflurane anaesthesia. For immunohistochemistry, muscles were fixed in 4% paraformaldehyde for 24 hours. After that, the whole muscle were cut transversely to muscle fibre into four slices with equal size. The slices underwent routine dehydration and were embedded in paraffin for further processing such as microtome sectioning and immunostaining.

### Study protocol

In series I, microvascular blood flow, relative hemoglobin amount and hemoglobin O_2_ saturation were determined spectroscopically in the injured area of the traumatized muscle and, for comparison, in the respective area of the contralateral non-traumatized muscle. Animals (n = 8) were ventilated with 100% O_2_. Measurements were performed before trauma (baseline), directly (1–2 minutes) after trauma and subsequently every 40 minutes. After 240 minutes, four of the animals were sacrificed. With the other four animals, measurements were continued for additional 240 minutes reaching a total observation time of 480 minutes before sacrifice.

In series II, hypoxia was estimated by immunostaining of pimonidazole and HIF-1α. The animals were divided into two trauma groups (IIa and IIb) and one control group without trauma (IIc). In the trauma group IIa (n = 16) and in the control group IIc (n = 6), animals were ventilated with 100% O_2_ (as in series I). In the trauma group IIb (n = 8), animals were ventilated with 21% O_2_. Thirty, 120, 210 and 450 minutes after trauma (group IIa) and 120 and 210 minutes after trauma (group IIb), respectively, the traumatized and the contralateral non-traumatized *Musculus gastrocnemius* were harvested. For this purpose, four of the animals were sacrificed at each time point. In the control group IIc, the right *Musculus gastrocnemius* was harvested 210 minutes after the initial preparation time of 60 minutes. In each group, pimonidazole (60 mg/kg body weight) was injected intravenously 90 minutes before harvesting the muscle.

### O2C measurements

**Figure 1:**
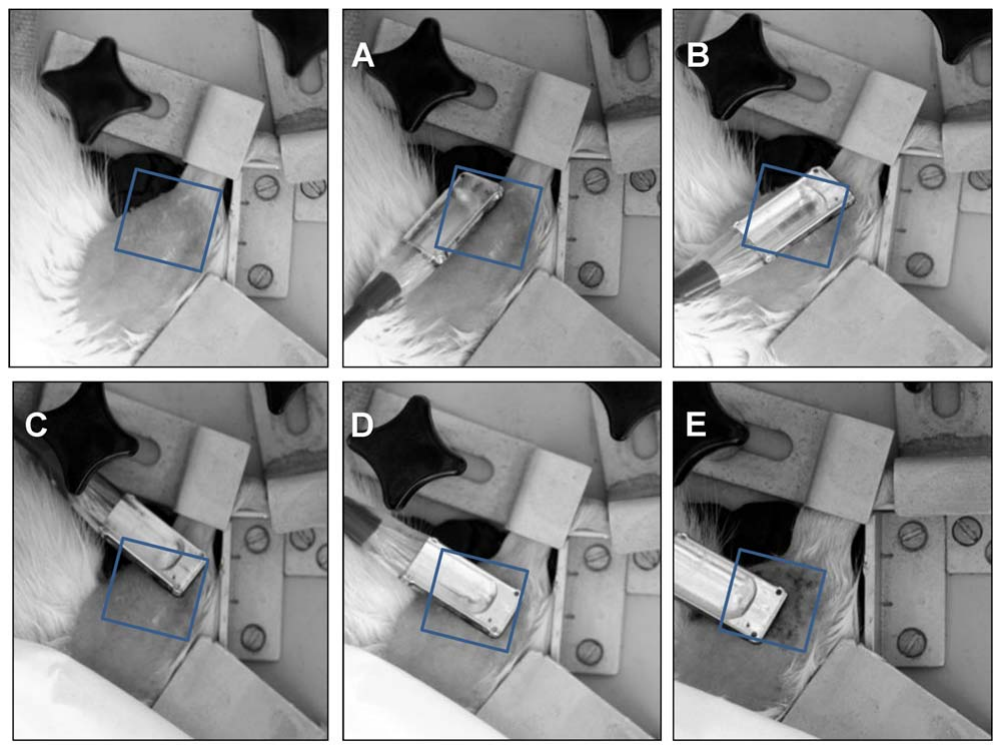
Probe positions at the injured area. Five different probe positions (A–E) were used for laser Doppler and white-light spectroscopic measurements with the spectrometer O2C (oxygen-to-see). The injured area of the dorsal compartment muscles of the shaved right lower hind limb is shown. A special glass fiber probe was used to collect data over 20 seconds at each position.

For non-invasive, local microvascular measurements the multiple channel system O2C was used. This equipment combines two different techniques to real-time analyze the microcirculation of the tissue: Laser Doppler and white-light spectroscopy [21]. Laser Doppler spectroscopy enables the measurement of microvascular blood flow. White-light spectroscopy allows to measure microvascular relative hemoglobin amount (rHb, microvascular blood filling) and microvascular hemoglobin O_2_ saturation (SO_2_). Measurements are confined to the microvascular system. The greater the amount of blood contained in the vessels, the more light will be absorbed by hemoglobin. Therefore, in larger vessels (>100 µm) light is absorbed completely and does not return to the detector system. Since the vast majority of the microvascular blood is in the capillary-venous system, the parameters measured mainly represent this vascular bed. A glass fibre probe (LF-2; LEA Medizintechnik, Germany) with a measurement depth of 4–6 mm and a surface diameter of 14 mm was used and placed consecutively on both shaved legs. The data were collected as a mean value over 20 seconds of measurement time at five different sites of the injured area (Figure 1).

### Immunohistochemistry pimonidazole

Immunohistochemical detection of covalently bound pimonidazole was performed according to the manufacturer's protocol for pimonidazole staining (Hypoxyprobe-^TM^1-Kit) with several modifications. In detail, the 4% paraformaldehyde-fixed paraffin-embedded blocks were cut into 2 µm thick transverse sections and mounted on poly-L-lysine-coated glass slides, which were dried overnight. At the beginning of the immunostaining, slides were deparaffinized with two 5-minutes rinses in xylene and rehydrated in a graded alcohol series (100%–75% ethanol) finally leading to water. To enhance antigen accessibility, heat-induced antigen-retrieval for 15 minutes was used. After this step and between all subsequent incubations, slides were washed three times with phosphate-buffered saline containing Tween 20 (PBST). Afterwards slides were exposed to 3% hydrogen peroxide for 10 minutes at room temperature (RT) to quench endogenous peroxidase activity. To prevent non-specific binding, serum-free protein block was used for incubation for 30 minutes at RT. Pimonidazole forms adducts with thiol-containing proteins at pO_2_ <10 mmHg. These adducts were detected with primary mouse anti-HP-1 (hpi Hypoxyprobe) at dilution 1∶50 in PBST and 1 drop DAKO protein blocker/ml for 40 minutes at RT. Thereafter slides were treated with Biotin-conjugated F(ab')2 fragment from a polyclonal rabbit anti-mouse IgG diluted 1∶500 in PBST and 1 drop DAKO protein blocker/ml for 10 minutes at RT, and were incubated with an immune peroxidase detection system, VectorstainABC kit, for 10 minutes at RT. 3,3′ diaminobenzidine, DAB was used to visualize the antigen-antibody by incubating for 15 minutes. After all, slides were washed with water and counterstained with hematoxylin for 45 seconds before being run through an alcohol dehydration gradient, cleared with xylene and then mounted with a coverslip and Crystal/Mount.

Traumatized muscle sections from animals that did not receive pimonidazole as well as sections stained without primary antibody were used as negative controls.

### Immunohistochemistry HIF-1α

Immunohistochemistry was performed on paraformaldehyde-fixed-paraffin-embedded samples according to the manufacturer's instructions (Vector Laboratories, USA; compare also Scheerer et al. [22]). As primary antibody a mouse anti-HIF-1α antibody (dilution 1∶10,000, Novus Biologicals) was used. For detection of HIF-1α a catalyzed signal amplification system (DAKO, Denmark) were used. Specific staining was visualized by incubation with 3′,3′-diaminobenzidine (Vector Laboratories). Hematoxylin was used for counter-staining.

### Analysis of pimonidazole staining and HIF-1α expression

For quantitative determination of the fraction of hypoxic cells, as indicated by immunohistochemistry, microscopic images from every slide of each muscle (traumatized and non-traumatized) were recorded. The hypoxic fraction (percentage area of pimonidazole-labelled muscle cells within the injured area) was determined by using a special Java-based image processing and analysis program (Image J, W. Rasband, National Institutes of Health, USA). Tissue regions showing pimonidazole staining were scored as positive, irrespective of staining intensity, and the remaining tissue was scored as negative.

For qualitative analysis of HIF-1α staining, the whole slides were examined and labelled cells scored as positive, irrespective of staining intensity.

### Statistical analysis

Statistical analysis was performed using SAS 9.4 (SAS Institute Inc., Cary, NC, USA). The data are displayed with mean ± SEM. The significance level was 0.05. To analyze series I, we used two-way repeated measurement ANOVA followed by Bonferroni post hoc analysis (between group differences). We did not test for pairwise within group differences because of lack of power (sample size n = 8), but we tested each time point to baseline for significance. All statements concerning within group differences are nevertheless descriptive. In series II, we compared each time point (n = 4) of the trauma group IIa with the control group IIc (without trauma, n = 6). For this, we used Bonferroni-corrected two-sample Wilcoxon tests.

## Results

### Biomonitoring

Mean arterial blood pressure and heart rate remained around 95 mmHg and 285 beats/min, respectively, throughout the experiment in all groups studied. The only exception was a slight, temporary increase in mean arterial blood pressure up to 105 mmHg directly after trauma.

### Microvascular blood flow, relative hemoglobin amount and hemoglobin O_2_ saturation

Measurements of microvascular blood flow (flow), relative hemoglobin amount (rHb), and hemoglobin O_2_ saturation (SO_2_) were performed directly in the injured area of the traumatized muscle and, for comparison, in the respective area of the contralateral non-traumatized muscle as shown in Figure 1.

**Figure 2:**
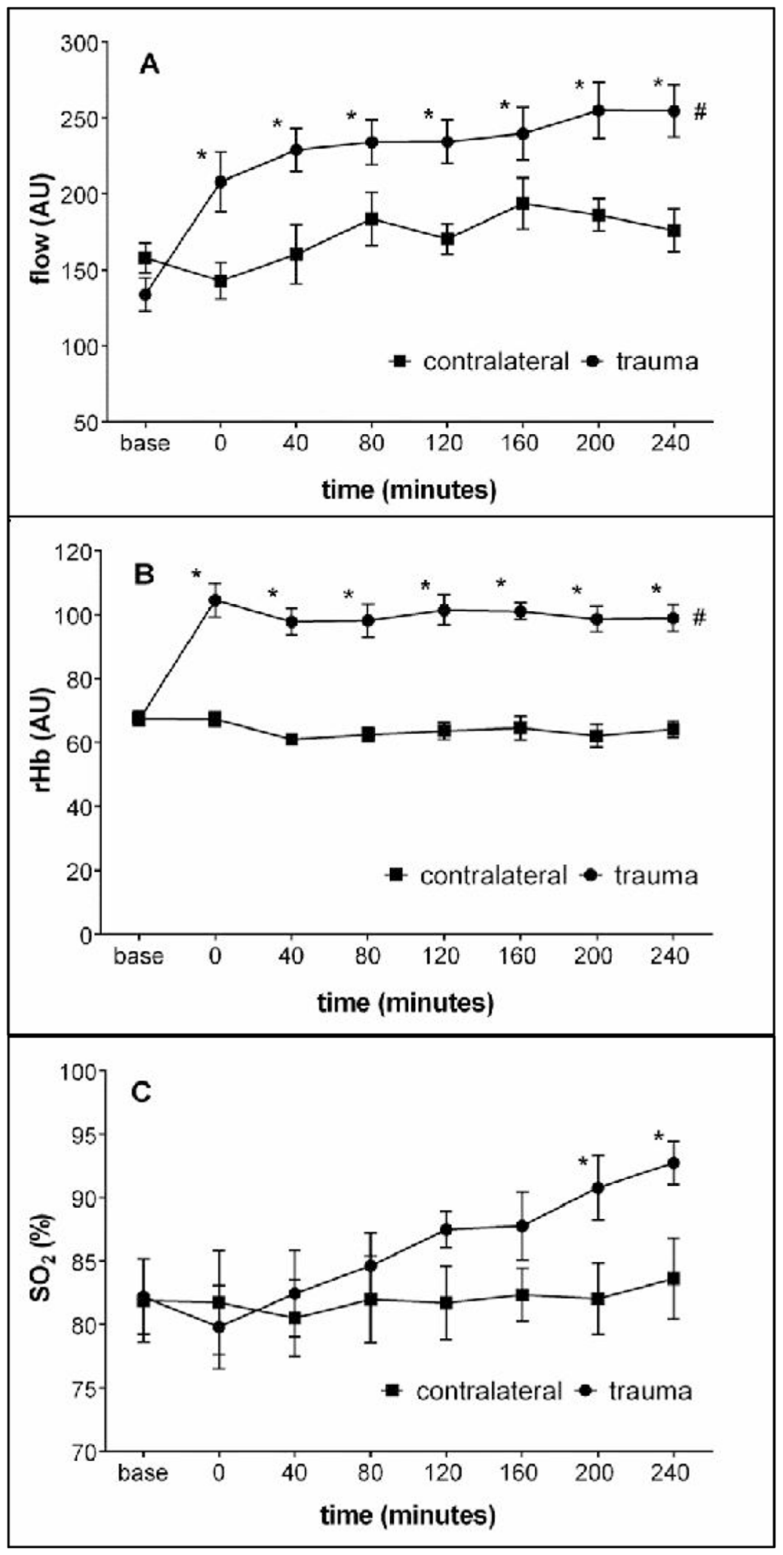
Effect of local blunt muscle trauma on microvascular blood flow, relative hemoglobin amount and hemoglobin O_2_ saturation. Animals (n = 8) were ventilated with 100% O_2_. Parameters were determined immediately before trauma (base values, base), 1–2 minutes after trauma (0) and every 40 minutes until the end of the experimental period. flow, microvascular blood flow (A); rHb, relative hemoglobin amount, representing blood filling of microvessels (B); SO_2_, microvascular hemoglobin O_2_ saturation (C); AU, arbitrary unit; trauma, injured area of traumatized muscle; contralateral, respective area of the contralateral non-traumatized muscle. Values shown represent means ± SEM. *P<0.05 (versus base). ^#^P<0.05 (versus contralateral; significant to all post trauma time points of flow and rHb except for flow at time point 160 minutes; not significant for SO_2_, but post-hoc t-tests reveal significant differences between the groups for the time points 200 and 240 minutes).

Immediately after trauma, blood flow in the injured area significantly increased (compared to base) from 134 AU to 208 AU. It continued to rise slightly up to 255 AU at 240 minutes after trauma and remained elevated around this value until the end of the experiment at 480 minutes (shown for the first 240 minutes in Figure 2A). Parallel to the rise in blood flow, there was an immediate significant increase from 67 AU to 104 AU in the relative hemoglobin amount, which persisted at this level for the whole experimental period (shown for the first 240 minutes in Figure 2B). In contrast to both other microvascular parameters, directly after trauma the microvascular hemoglobin O_2_ saturation was similar to baseline values (approximately 80%). However, 200 minutes after trauma, the microvascular hemoglobin O_2_ saturation reached a significant increase (to 91%) and remained elevated until the end of experimental period (shown for the first 240 minutes in Figure 2C).

In the contralateral non-traumatized muscle, microvascular blood flow, relative hemoglobin amount and hemoglobin O_2_ saturation remained constant throughout the whole experimental period until 480 minutes after trauma (shown for the first 240 minutes in Figure 2).

The difference in traumatized and contralateral non-traumatized muscle was significant for flow and rHb, but not for SO_2_.

### Pimonidazole staining

**Figure 3:**
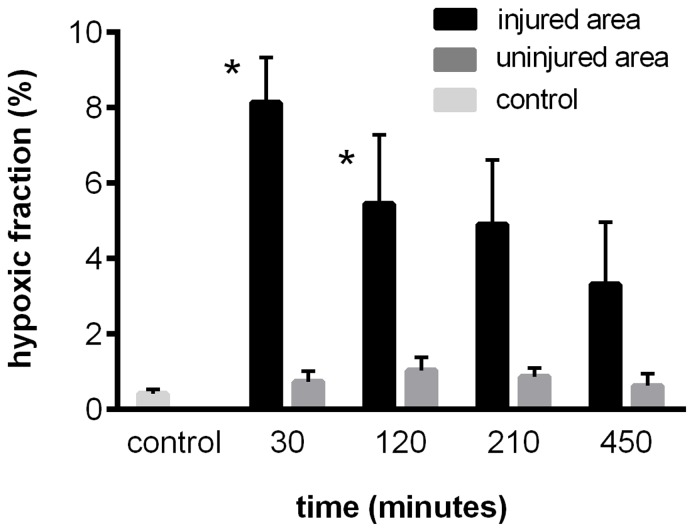
Hypoxic fraction as indicated by pimonidazole staining. Animals were ventilated with 100% O_2_. The traumatized *Musculus gastrocnemius* of the trauma group IIa (n = 4 for each time point) and the *Musculus gastrocnemius* of the control group IIc (n = 6) were harvested and sections of the muscle specimen were analyzed for pimonidazole binding. Hypoxic fraction, percentage area of pimonidazole-labelled muscle cells within an area; injured area, injured area of the traumatized muscle of the trauma group IIa; uninjured area, the adjacent uninjured area of the traumatized muscle of the trauma group IIa; control, tissue area of the non-traumatized muscle of the control group IIc. Values shown represent means ± SEM. *P<0.05 (versus control).

As indicated by pimonidazole staining (Figure 3), there was hardly any hypoxia in the muscle of the control group (group IIc, animals ventilated with 100% O_2_). In the trauma group IIa (animals likewise ventilated with 100% O_2_) 30 minutes following trauma, about 8% of the injured area were hypoxic (significantly different compared to control group). Subsequently, the hypoxic fraction in this area somewhat decreased reaching 3% 450 minutes after trauma (no longer significantly different from the control group). In the adjacent uninjured area of the traumatized muscle less than 2% of hypoxic fraction was found at each time point.

Decrease of the O_2_ content of the inspired air from 100% to 21% (group IIb) had no effect on the hypoxic fraction as determined at 120 (3.3%±0.4) and 210 minutes (2.8%±0.5) following trauma (data not shown).

**Figure 4:**
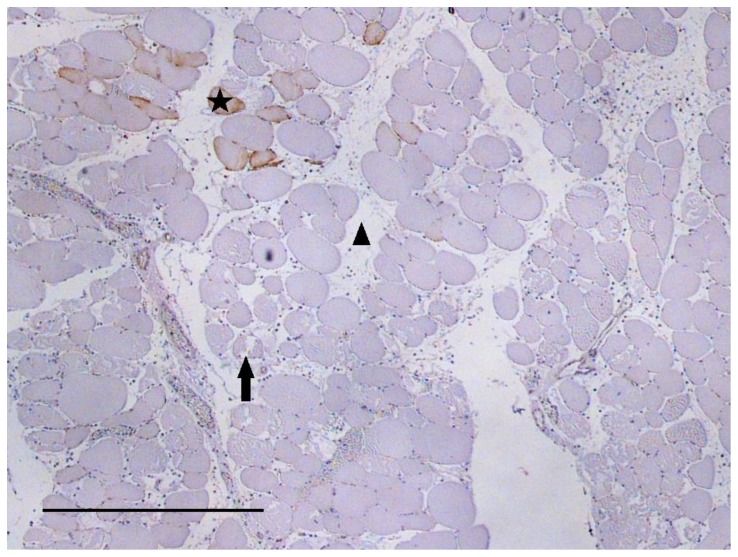
Pimonidazole staining within injured muscle area (representative figure). The animal was ventilated with 100% O_2_. 450 minutes after trauma, the *Musculus gastrocnemius* of the traumatized right hind limb was harvested and section of the muscle specimen was analyzed for pimonidazole labelling within the injured area. Injured area is identified by e.g. necrotic muscle cells (arrow) and edema (arrowhead). Hypoxic muscle cells indicated by pimonidazole binding are stained brown (star). Scale bar: 500 µm.

At each time point of the trauma groups IIa and IIb, the hypoxic cells were either located separately or located in groups of up to 7 cells, evenly distributed over the whole injured area (Figure 4).

Traumatized muscle sections from animals that had not been treated with pimonidazole and of sections of pimonidazole-treated animals where the primary antibody was omitted during the staining procedure served as negative controls, and did not show any pimonidazole labelling.

### HIF-1α expression

**Figure 5:**
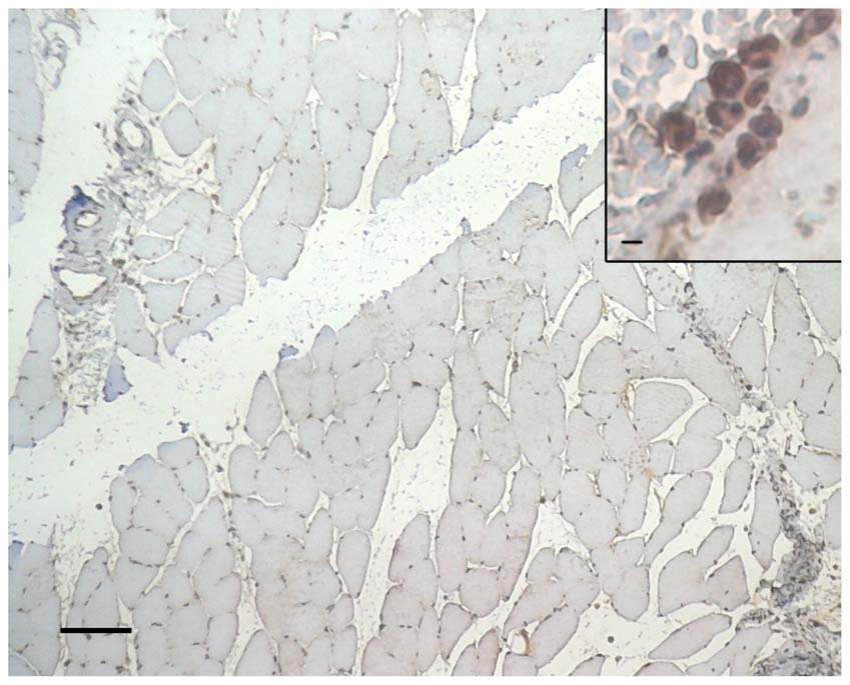
HIF-1α staining within injured muscle area (representative figure). The animal was ventilated with 100% O_2_. 450 minutes after trauma, the *Musculus gastrocnemius* of the traumatized right hind limb was harvested and section of the muscle specimen was analyzed for HIF-1α expression within the injured area. No significant staining for HIF-1α of the muscle cells. Scale bar: 150 µm. Inlet: Positive staining for HIF-1α expression by myeloid cells (brown), invading to the traumatized muscle. Scale bar: 10 µm.

In skeletal muscle cells there was hardly any positive HIF-1α-staining. This was also true following trauma (Figure 5), where no significant alteration in the number of HIF-1α positive skeletal muscle cells was detectable at each time point studied (30, 120, 210 and 450 minutes after trauma). Only myeloid cells, invading to the traumatized muscle, stained positive for HIF-1α (Figure 5, inlet). Accordingly, positive staining for HIF-1α increased with numbers of invading myeloid cells which was minimal at 30 min after trauma and became substantial at 120, 210 and 450 minutes after trauma.

## Discussion

The combination of laser Doppler with white-light spectroscopy as applied in the spectrometer O2C (oxygen-to-see) provides information about microcirculatory alterations and O_2_ supply in well-defined tissue areas. In the present experiments, the device was used to follow these parameters in the dorsal compartment muscles of the right lower hind limb after severe blunt muscle trauma. The results indicate a clearly raised microvascular blood flow, a considerably enhanced microvascular relative hemoglobin amount and a distinct elevation of the microvascular hemoglobin O_2_ saturation (Figure 2). Together, these data strongly suggest a largely increased O_2_ supply to the injured muscle area. Comparable results, although less pronounced, i.e. a markedly raised microvascular blood flow but unaltered relative hemoglobin amount and hemoglobin O_2_ saturation, have recently been published by Rotter et al. [11]; in their experiments the dorsal compartment muscles of the left lower hind limb of rats were injured by a blunt high-velocity trauma.

An enhanced microvascular perfusion of the injured area and an enhanced O_2_ supply to this region also readily account for an increased macrovascular blood supply to the traumatized muscle and a raised O_2_ partial pressure of the venous blood draining the injured tissue as has been shown for the external iliac and femoral artery and the femoral vein, respectively, after muscle trauma of the lower hind limb [14]–[17].

In our experiments the mean arterial blood pressure remained almost constant and since there was no change in the microvascular perfusion of the contralateral non-traumatized muscle, mediators released locally in the injured tissue should be responsible for the enhanced perfusion of the traumatized muscle. As the rise in microvascular blood flow within the injured area occurred instantaneously, at least some of the mediators being responsible for the hyperemia need to be released immediately after trauma. The exact nature of these mediators especially after muscle trauma, however, is unknown at present. Possible candidates are nitric oxide (NO), adenosine triphosphate (ATP), potassium (K^+^), histamine and prostaglandins such as prostacyclin [16], [23].

There is no doubt that within the injured area at least in some of the microvessels perfusion is impaired, e.g., due to mechanical destruction of these vessels. Accordingly, in intravital fluorescence microscopy studies of the traumatized muscle, significant reduction in functional capillary density could be observed [6]–[11]. Based on this observation, an impairment of nutritive perfusion, resulting above all in a limitation of O_2_ supply, is generally assumed [1]–[6]. The results of the spectroscopic microvascular measurements we obtained in our study, however, cast severe doubt on this assumption. They strongly suggest that an increased perfusion of residual intact microvessels is obviously capable of compensating or even overcompensating the deficit in nutritive perfusion. In line with this consideration, an increased postcapillary blood flow and an increased capillary diameter (especially venular diameter) have been reported in some of the intravital fluorescence microscopy studies [7], [10], [11]. However, in spite of the overall increased microvascular blood flow, inhomogeneous perfusion caused by a redistribution of blood flow may result in the coexistence of hyper- and hypoperfused vessels, a phenomenon which is known as “functional shunting” and which has also been described to occur after soft tissue trauma [24]–[26].

Although the spectroscopic microvascular studies did not provide any evidence for an impaired O_2_ supply and thus hypoxia within the injured muscle area, but suggest an even increased O_2_ supply instead, hypoxia may still exist at the microscopic (cellular) level, e.g., due to inhomogeneous perfusion (see above). To verify this possibility, we applied pimonidazole hydrochloride (Hypoxyprobe™-1), a 2-nitroimidazole derivative forming covalent adducts with thiol groups in proteins, peptides and amino acids under hypoxic conditions (O_2_ partial pressure <10 mmHg). Pimonidazole has been used in numerous studies before, particularly in cancer research but also in studies on different kinds of wound healing [27]–[30]. In line with results of recent experiments performed with mice [22], staining with pimonidazole indeed revealed a significant number of hypoxic cells following trauma, being present alone or in groups of a few (2 to 7) cells evenly distributed over the whole injured area (Figure 4). Thus, presumably due to impaired perfusion of some of the microvessels, at the cellular dimension distinct areas of hypoxia exist in the traumatized muscle. These areas may also account for a slight increase in NADH fluorescence and a decrease of reduction of triphenyltetrazolium chloride (TTC), which has been reported to be detectable in the traumatized muscle and which indicates a limitation of O_2_ supply of cytochrome oxidase of the mitochondrial respiratory chain [9], [11]–[13]. On the other hand, however, the number of hypoxic cells clearly remained low (hypoxic fraction of 8%, 30 minutes after trauma), in addition, without any tendency to increase with time (Figure 3). Therefore, the vast majority of muscle cells within the injured area either remains under normoxic conditions or should even be confronted with a surplus of O_2_.

Hypoxia-inducible factor 1 (HIF-1) is a transcription factor that regulates the transcription of hypoxia-inducible genes, mediating a variety of essential cellular responses to hypoxia. HIF-1 consists of a hypoxia-inducible α-subunit and a constitutively expressed β-subunit localized to the nucleus. Under hypoxia, HIF-1α protein is stabilized, accumulates, and is then translocated into the nucleus, where it dimerizes with the β-subunit [31]. In the present experiments marginal expression of the HIF-1α subunit by skeletal muscle cells was detected, not only under control conditions but also in the traumatized muscle, thus providing no evidence for the occurrence of hypoxia in the traumatized muscle and further supporting the conclusions drawn from pimonidazole staining. In contrast to the skeletal muscle cells, however, there was a definitive expression of HIF-1α by invading inflammatory cells (Figure 5), in line with recent results of Scheerer et al. [22] who have already shown that myeloid cells invading to mouse traumatized muscle stained positive for HIF-1α. Since the expression of the HIF-1α subunit can be increased by inflammation, either by inducing transcription or translation depending on the type of inflammatory stimulus [32], the present results indicate that the expression of HIF-1α in these cells is rather due to inflammatory stimuli than due to hypoxia.

In mechanical tissue injury, hypoxia is assumed to decisively contribute to additional injury to otherwise uninjured cells, known as secondary injury [33], [34] and later to limit healing of the lesion [3]. According to the present results, however, hypoxia should only be of minor importance for secondary lesion growth and also not decisively compromise healing of the injured area. Instead, one may even speculate that the surplus supply with O_2_ may represent an even greater injurious burden to the already mechanically injured tissue than hypoxia. The increased perfusion of large parts of the injured muscle area should also facilitate invasion of inflammatory cells, which may also enhance secondary injury of the injured area but, on the other hand, may also accelerate healing [35], [36].

Our study has some limitations. The experiments were performed with animals anaesthetized with isoflurane and ketamine. Isoflurane is usually assumed to induce peripheral vasodilation and thus to increase muscle blood flow whereas ketamine can cause arteriolar vasoconstriction and thus a decrease in blood flow [37]–[39]. However, trauma was induced in the present experiments with a delay of 60 minutes after the introduction of anaesthesia. Thus, the observed alteration of perfusion immediately after trauma should rather be a direct consequence of the trauma than a reaction to the anaesthetic agents.

In our hands, ventilation with 100% O_2_ is required to perform experiments with the anaesthetized animals lasting longer than 210 minutes. Within the first 210 minutes after trauma, however, basically the same results were obtained with animals breathing 21% O_2_. Furthermore, a high O_2_ content of the inhaled air does not necessarily result in a higher O_2_ partial pressure within the muscle tissue [40]. Thus, it is justified to assume that the O_2_ conditions in the injured muscle area as described above also apply to normal air breathing animals.

Due to the experimental approach chosen, our results are only valid for the early phase after tissue injury of up to 8 hours. This time window already covers important processes of secondary injury [13], [41], but crucial injurious and healing processes accompanied by ischemia/hypoxia may still occur at later time points. For instance, maximal swelling takes place up to 72 hours following muscle trauma [7], which may result in profound ischemia of the affected limb.

It is important to note that the conclusions drawn so far are only applicable to blunt muscle trauma without violation of larger blood vessels and without overt systemic reactions. Within these constraints, however, the model chosen produces a comparatively quite severe injury [6], [39], [42]. It is obvious, that less severe forms of blunt muscle trauma should result in even less hypoxic areas. On the other hand, however, in other forms of muscle trauma such as missile trauma and trauma with disruption of larger blood vessels or under conditions of shock, the hypoxic fraction within the injured area may be largely increased [43].

It should also be noted that the (resting) muscle may behave differently from other tissues with respect to hypoxia following trauma, amongst others, due to its high vascular density. For instance, in closed muscle trauma usually large hypoxic areas in the skin lying above the traumatized muscle can be found [8], which was also observed in the present experiments (unpublished results).

Although several additional experiments are required, especially with respect to longer time courses and awake, non-anaesthetized animals, and although the results need to be verified clinically, it is conceivable that on their basis new treatment strategies can be developed: Due to the increased perfusion, there should be no problem to bring drugs directly into the injured area, an option, which may be used for instantaneous intravenous therapy after trauma. In this context, it might be considered to expand the spectrum of possible therapeutic agents by compounds targeting O_2_-dependent (oxidative) tissue injury.
